# Evaluation of exosome derivatives as bio-informational reprogramming therapy for cancer

**DOI:** 10.1186/s12967-021-02768-8

**Published:** 2021-03-09

**Authors:** Michael J. Gonzalez, Mercedes F. Kweh, Pier Mario Biava, Jose Olalde, Alondra P. Toro, Pascal J. Goldschmidt-Clermont, Ian A. White

**Affiliations:** 1 Medical Sciences Campus, School of Public Health, University of Puerto Rico, San Juan, Puerto Rico; 2 School of Medicine, Chiropractic Program, Universidad Central del Caribe, Bayamon, Puerto Rico; 3 Neobiosis, LLC, UF Innovate Biotech Building, Research Drive, Alachua, FL 12085 USA; 4 Via Milanese, 300, 20099 Sesto San Giovanni, MI Italy; 5 Centro Medicina Regenerativa (CMR), Bayamon, Puerto Rico; 6 Department of Biology, University of Puerto Rico, Mayagüez Campus, Mayagüez, Puerto Rico; 7 Alzady International, LLC, 3041 Orange St, Miami, FL USA

**Keywords:** Exosomes, Cancer, Cytosomes, Signaling, Reprogramming, Therapy

## Abstract

Exosomes are nanoparticle sized (100 ± 50 nm) extracellular vesicles (ECVs) that play important roles in cell-to-cell communication. They do this by utilizing their natural ability to shuttle signaling molecules across the cellular microenvironment and promote paracrine signaling. Currently, exosomes are being explored for their potential as therapeutic agents for various degenerative diseases including cancer. The rationale behind their therapeutic ability is that they can transfer signaling biomolecules, and subsequently induce metabolic and physiological changes in diseased cells and tissues. In addition, exosomes can be used as a drug delivery system and may be very effective at reducing toxicity and increasing bioavailability of therapeutic molecules and drugs. Although exosomes were first believed to be a waste product of the cell, current research has demonstrated that these particles can serve as modulators of the immune system, act as cancer biomarkers, cause re-differentiation of cancer cells, and induce apoptosis in diseased cells. Extensive research has been performed specifically using amniotic fluid-derived extracellular vesicles, named “cytosomes”. While the use of cytosomes in clinical application is still in the early stages, researchers have shown great potential for these EVs in regenerative medicine as immune modulators, in controlling microbial infection and by inducing tissue repair through the activation of endogenous, tissue-specific stem cells. This review emphasizes the capabilities of specific subsets of extracellular vesicles that can potentially be used for cancer therapy, principally as a source of bi-informational reprogramming for malignant cells.

## Introduction

Back in 2018 it was estimated that over 18.1 million new cancer cases will arise in 2019 and 9.6 million cancer deaths will result [1]. With advanced age, cancer has become a pandemic disease, a global scourge. Despite some significant therapeutic advancements in recent decades, clinical outcomes of most cancers remain poor [2]. In an attempt to solve this major issue, new therapeutic avenues are eagerly sought. As such, we would like to promote exosomes as possible anticancer tools. Exosomes, which were originally described in 1983 [3], are small extracellular vesicles (EVs) that are secreted by most cell types including, endothelial cells, epithelial cells, MSCs, stem cells, platelets and tumor cells [4]. They are nano-sized lipid membrane-bound vesicles that are ubiquitously found in biological fluids such as tears, plasma, urine, saliva, breast milk, amniotic fluid, cerebral and synovial fluids [5]. Exosomes are produced through invagination of the cellular membrane and then released from the cell via exocytosis. Exosome biogenesis begins with late endosome budding inward forming intralumenal vesicles (ILV) that accumulate and result in the formation of multivesicular bodies (MBV) [6]. These MVB can either become degraded within lysosomes or move to the plasma membrane where they fuse with the plasma membrane and release endosomes from the cell surface. The endosomal vesicles released are between 50–250 nm in diameter, depending on which cell type they originate from, and have uniform topology [6].

Invagination results in exosomes with cargo reflective of their cellular origin and consequently the molecular content of each may be used as disease biomarkers, most notably in cancers [4]. During the process of invagination proteins get integrated into the newly formed ILV. In addition, cytosolic components are also enclosed within the ILV. As a result of the process of their formation, exosomes contain unique cargo signatures. Currently, there are over 40,000 entries, collected from 286 studies, of exosomal cargo described on ExoCarta (Version 4; http://www.exocarta.org). ExoCarta reports 9769 unique proteins that have been identified within exosomes. While these protein profiles change dependent upon the cell of origin, there remain a number of proteins that are consistently found within exosomes, and these may be leveraged as exosome-specific biomarkers.

Evidence from recent reports suggests that ILV formation is dependent upon the endosomal sorting complex (ESCRT) [6]. The function of ESCRT is believed to include the formation of MVB, membrane budding and protein sorting. In addition to the four ESCRT (ESCRT-0, -I, -II and -III) proteins, other typical exosomal proteins, that are known to aid in biogenesis, include Alix, Vps4 and other ESCRT proteins (TSG101 and CHMP4) [6]. Currently, the most commonly used biomarker proteins are the tetraspanins (CD82, CD81, CD63, and CD9), heat shock proteins (HSP70, HSP90) and proteins involved in membrane transport and fusion (annexins and Rab) [6].

There is emerging evidence to indicate that the molecular content of exosome cargo, which includes miRNAs, mRNAs, proteins, complex sugars and lipids, allows exosomes to play a significant role in paracrine signaling [2, 7]. Exosomes circulate systemically throughout the body and carry these molecules from signaling to target cells. Once they reach the target tissue, they induce various biological activities, including immune modulatory responses, intercellular transport and communication, metastasis (in the exclusive case of caner), angiogenesis and cellular survival [8, 9, 56].

Given these characteristics, considerable research is currently underway to explore the potential of exosomes in the study of human cancers. Exosomes, derived from cancer cells contain specific cancer biomarkers, which can be used in early diagnosis and prognosis. Furthermore, in the context of cancer immunotherapy, exosomes have demonstrated promise since they express the necessary immunogenic antigens to stimulate NK cells and anti-tumor T-lymphocytes [10]. Herein, we propose that amniotic fluid-derived EVs (termed "cytosomes” in a recent review by Dr. Ian White) [11], may provide the cellular signals necessary for malignant cells to re-differentiate or undergo programed cell death (apoptosis) and are therefore a potential therapeutic tool in the fight against malignant tumors. We believe that the reprogramming/re-differentiation signals delivered by exogenous cytosomes, are capable of suppling the necessary reprogramming signals to overcome malignant cellular commitment, and therefore, restore normal cellular biological behavior in previously transformed cells.

## The potential for using oncosomal-derived biomarkers in cancer diagnosis and prognosis

Scientists and health professionals are harnessing the potential of exosomes as biomarkers for the diagnosis of disease. Studies have demonstrated that cancer patients display an array of unique exosome sub-types (collectively called "oncosomes"), and these oncosomes are elevated in the systemic circulation of patients with cancer [12]. Consequently, oncosomes can be used in cancer diagnosis, and likely utilized at earlier stages compared to canonical cancer biomarkers. Assessment of oncosome cancer biomarkers is achieved by harvesting a sample of biological fluid from a patient, followed by isolation of the oncosomes produced by the cancer cells. A number of methods are available for the isolation EVs from biological fluids. The current gold-standard in the field of exosome research, accounting for over 50% of all isolations, is differential ultracentrifugation [13].

Blood is the most abundant source of oncosomes in the body and is therefore used most for their isolation [7]. In 2013, Tanaka et al. isolated oncosomes from serum samples of patients with esophageal squamous cell cancer (ESCC) [14]. This study revealed that patients with more severe ESCC, which presented with systemic inflammation, expressed significantly higher levels of oncosomal miR-21 compared to patients with more benign disease [14]. The authors found that miR-21 was not only positively correlated with tumor progression, as it was associated with metastasis and lymph node status, but also with tumor aggressiveness, since it was found primarily in the most advanced stages disease [14]. More recently, Liang et al. isolated miR-223 from platelet-derived microvesicles of patients with non-small cell lung cancer (NSCLC) [15]. They concluded that miR-223 was essential in promoting tumor cell invasion in NSCLC patients by targeting EPB41L3. Subsequently, they were able to largely abolish invasion by targeting miR-223 and/or downregulating EPB41L3 [15]. This strongly indicated that mir-223 could be useful as a potential biomarker for NSCLC and could be used to create novel cancer treatments. Oncosomes were first characterized in 2008 by Al-Nedawi et al. [16]. This group discovered that the oncogenic form of the epidermal growth factor receptor (EGFRvIII), which causes oncogenesis by activating the signaling pathways MAPK and Akt and regulating gene expression of VEGF, Bcl-x(L) and p27, was actively transferred via microvesicles termed “oncosomes” between glioma cells resulting in their transformation [16]. In terms of the specific characteristics of oncosomes, which may differentiate them from other EVs, the potential to transform naïve cells into oncogenic cells is the most profound [17]. Morphologically and phenotypically their specific characteristics are dependent upon the cell type of origin and the particular oncogenic related molecules they carry, while in general they are described as a population of large EVs, with "a round cup-shaped morphology", roughly 1–10 um in diameter [18]. Because of their larger relative size, oncosomes exhibit a distinct buoyant density between 1.10 and 1.15 g/ml and can be isolated by floating pellets on discontinuous density gradients [19, 20]. In addition, cancer related proteins are observed to be associated with oncosomes, including enzymes involved in glucose, glutamine and amino acid metabolism with cytokeratin being the most abundant protein found [21]. Specific uniquely expressed proteins have also been reported to be expressed in large and nano-sized EVs including, LAMB2, MSLN, GPR126, ITGA6, PYCRL, ITGA5 and GLG1 [21].

Oncosomes may open a new avenue for improved diagnosis of cancers since they exhibit the potential not only to help in diagnosis, but can also inform the physician on tumor status, which would be critical in determining appropriate treatments (Table 1). A recent study by Wong and Chen assessed the clinical significance of exosomes in cancer diagnosis and prognosis [40]. They conducted a meta-analysis evaluating diagnosis from 47 biomarkers and 2240 patients from 30 studies; also evaluating prognosis from 50 biomarkers and 4797 patients from 42 studies using QUADAS-2 and REMARK. Biomarkers included in the study were miRNAs, lncRNAs and proteins from a wide range of cancers. Respectively, the resulting specificity and sensitivity for each cancer diagnostic marker analyzed were: colorectal cancer 0.87 and 0.57, gastric cancer 0.73 and 0.77, pancreatic cancer 0.90 and 0.91, liver cancer 0.80 and 0.76 and prostate cancer 0.79 and 0.77 [40]. Similarly, this group assessed the summary receiver operating characteristic (SROC) curve and plotted the area under the curve (AUC) calculating the Q* index which they used to estimate the overall diagnostic performance. The following AUC and Q* resulted from their analysis of each disease studied: colorectal cancer 0.88 and 0.82, gastric cancer 0.84 and 0.77, pancreatic cancer 0.93 and 0.87, liver cancer 0.84 and 0.77 and prostate cancer 0.86 and 0.79, with an AUC of 0.5 suggesting (no diagnostic ability), 0.7–0.8 (acceptable), 0.8–0.9 (excellent), and 0.9–1.0 (outstanding), together these data suggests that oncosomal biomarkers have great potential as a diagnostic tool [40]. A liquid biopsy (blood samples and others) containing oncosomes can easily supplement the current armamentarium of cancer biomarkers and possibly provide a more suitable and non-invasive procedure for patients, and as such they warrant future study as novel therapeutic agents. Importantly we should advise the reader that “oncosomes” are derived specifically from cancer cells, and thus contain cargo to favor unchecked mitosis and metastasis. They are to be distinguished from healthy exosomes (cytosomes), which may contribute to re-differentiation/reprograming, as previously discussed. In this review we propose two ideas pertaining to the use of exosomes in cancer diagnosis and treatment. The first is that *oncosomes* may be a novel and effective tool in cancer diagnosis/prognosis. The second is that *cytosomes*, and other EVs, may be effective tools with the potential as novel anti-cancer therapeutics.

**Table 1:** Isolated and characterized oncosomes in cancers

Disease or cell type	References
Glioblastoma cancer cells	[18]
	[22]
	[23]
	[16]
Breast cancer	[24]
	[25]
Prostate cancer	[26]
	[27]
	[28]
	[29]
	[30]
Skin cancers	[31]
Urologic cancers	[32]
Oral cancers	[33]
Lung cancers	[34]
Gastrointestinal cancers	[35]
	[36]
Head and neck cancers	[37]
	[38]
	[39]

## The roles of exosomes in cancer therapy

### Mechanistic role of exosomes in cancer

Exosomes have been proposed to play a major role in epithelial to mesenchymal transition (EMT) and mesenchymal to epithelial reverting transition (MErT) [41]. During EMT tumor cells obtain mesenchymal characteristics and initiate the metastatic process, decreasing proliferation and increasing migration and invasion. This state is associated with the zinc finger proteins Snail and Slug, zinc finger and homeodomain proteins Zeb1 and Zeb2 and the helix-loop-helix TWIST proteins [41]. Some exosomes found in cancer cells express high levels of HIF1α (hypoxia-inducible factor 1α), which interacts with the Snail pathway and increases TWIST expression resulting in the initiation of EMT [42]. Increased expression of the mesenchymal marker vimentin and decreased expression of the epithelial markers E-cadherin and β-catenin are typical during EMT. Exosomes have been shown to promote induction of vimentin and the inhibition of E-cadherin and β-catenin [41], which in the case of oncosomes can result in increased MErT and the initiation of metastasis [41].

### Exosomes as drug and molecular carriers

Exosomes function as superior drug delivery tools in cancer therapy owing to their very small size, minimal toxicity, capacity for intercellular transport and immune evasion. For these reasons, exosomes constitute excellent drug and molecular carriers and have the potential for use in small molecule therapy for targeted drug delivery to cancer cells. One of the most desirable approaches in cancer therapeutics is a targeted specificity to malignant cells, with minimal "off target" toxicity to surrounding cells, tissues and organs. The ability of exosomes to express targeting molecules with tropism for cancer cells may be an essential characteristic, which revolutionizes target specificity in cancer treatment [43]. This homing ability allows for drug and molecular payloads to be delivered directly into oncogenic cells, bypassing healthy neighboring cells. Presently, there exist several techniques by which therapeutic molecules can be loaded into exosomes, with sonication and electroporation being among the most popular [44]. Exosomes as a delivery system can be highly advantages in its potential to phenotypically modify cells. However, some criteria should be taken into consideration when using exosomes to deliver cargo such as its source, methods of extraction, purification and storage of exosomes should also be critically considered and optimized before clinical use [44].

The use of exosomes as a nanoparticle drug delivery system has gained significant interest in recent years. In 2016, researchers packaged exosomes with curcumin [45], which is an anti-inflammatory agent found in *Curcuma longa,* and has previously been used in the treatment of certain cancers [46]. However, simple supplementation of curcumin alone results in limited efficacy due to a lack of bioavailability [47]. In their published research Anand and colleagues demonstrated that exosomes isolated from H1299 cells, pretreated with curcumin, exhibited substantial anti-cancer effects through the upregulation of TCF21 [45]. Moreover, they explained that as curcumin was packaged within exosomes, the bioavailability in patients was significantly increased leading to enhanced therapeutic effects.

Current standard of care using chemotherapy has been criticized for the sever toxicity and harsh side effects experienced by patients. Doxorubicin is a chemotherapy drug approved by the FDA, which is commonly used due to its efficacy in fighting multiple types of cancer with acceptable response rates and overall survival [48]. However, this drug is known to cause apoptosis and necrosis in healthy cells and organs [49]. Research conducted in 2014 tested the potential of exosomes as a drug delivery system for doxorubicin [50]. The results demonstrated that when doxorubicin was loaded into exosomes cell proliferation was inhibited in various tumor cell lines more effectively than when doxorubicin was administered alone. Critically, the targeting ability of exosomes resulted in less off-target toxicity compared to systemic administration of doxorubicin alone. A subsequent study looking at doxorubicin and paclitaxel identified the importance of exosome carrier therapy for therapeutic delivery through the Blood Brain Barrier (BBB) [51]. Both large and small molecular anti-cancer treatments experience decreased efficacy due to their inability to cross the BBB. However, Yang et. al used exosomes to deliver doxorubicin and paclitaxel across the BBB and into the brain of a zebrafish, which resulted in fewer viable cancer cells compared to the drugs alone [51].

Several published reports have also described the use of exosome carriers in the delivery of nucleic acid molecules, as such as siRNAs, miRNAs and ssDNAs [52]. Small nucleic acid molecules have the potential for therapeutic use, but their inability to cross cell membranes and the disposition of RNA for rapid degradation make them difficult to effectively administer clinically. Lamichhane et. al successfully used exosomes to deliver an siRNAs cargo into H293T cells, which robustly reduced expression of the oncogenic tyrosine kinase HER2 [53]. Targeting exosomes to specific cancer cells can be enhanced by engineering the exosome to express certain peptides on their surface. Ohno et. al engineered exosomes to express the peptide GE11 to efficiently deliver miRNA to epidermal growth factor receptor (EGFR)-expressing breast cancer cells [54]. These data, taken together, identify a major role for exosomes as an efficient drug delivery system, providing enhanced drug delivery with fewer off-target effects.

### Exosomes as immune enhancers

**Fig. 1:**
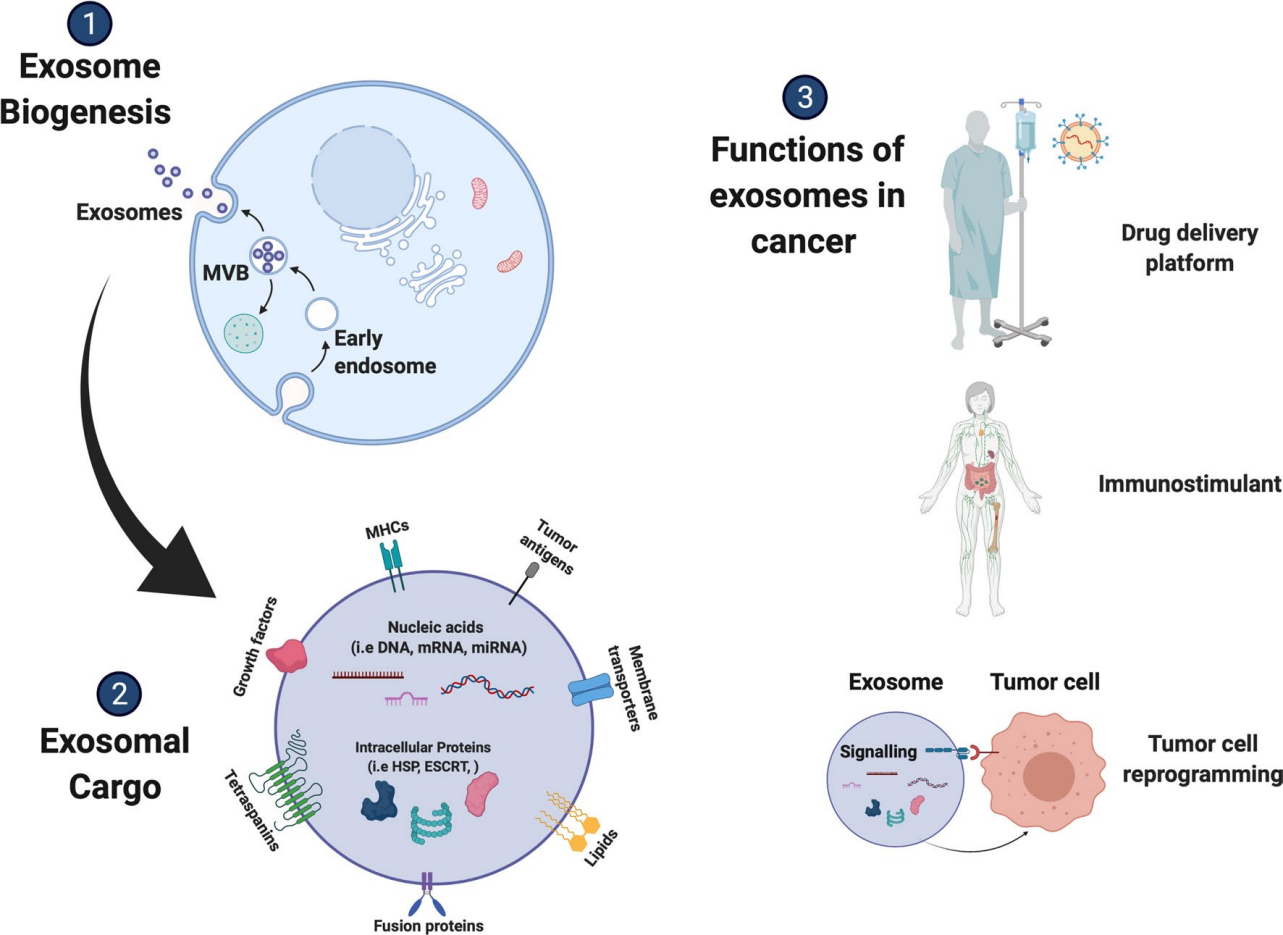
The main functions of exosomes in cancer. Exosome biogenesis results from inward budding forming intralumenal vesicles that accumulate and result in the formation of multivesicular bodies (MVB). The MVB then moves to the plasma membrane, fuses and releases endosomes. As a result of the process of their formation, exosomes contain unique cargo signatures which include proteins, nucleic acids, growth factors, tetraspanins and lipids. Because of their natural ability to shuttle signaling molecules, these EVs have the ability to be engineered to contain specific cargo to be used as drug delivery platforms for treating disease. They also have an excellent capacity as immune modulators and can send signals to reprogram malignant cells

When exosomes were first studied they were believed to be aggregates of cellular waste. Subsequent research uncovered a more multifarious role in direct cell-to-cell communication [55]. This function forms a critical component in modulating the immune system and other biological processes [56]. Immunotherapy is a treatment that stimulates the immune system to fight more effectively against cancer (Fig. 1). In recent years, exosomes have been experimentally deployed as a novel cancer immunotherapy [1–3]. Tumor cells are known to release specific types of exosomes, which have been implicated in the regulation of the adaptive immune system and aid in immune avoidance [5]. Conversely, cancer-derived exosomes can also express MHC class I molecules and be a source of tumor antigens that T-cells subsequently recognize [57]. These molecules are very important for the stimulation of immune cells that can help in tumor growth inhibition. Moreover, tumor inhibition has been detected after in vivo administration of exosomes expressing tumor antigens. The mode of action may involve stimulation of CD4 + and CD8 + T cells [10], which are known to cause apoptosis in tumor cells [7].

### Exosomes as cellular modulators and biological re-programmers

The composition of exosomes has received much study in recent years. Thousands of different proteins have been identified as cargo packaged within exosomes (Fig. 1). Specific nucleic acids like mRNA have also been found within exosomes and are able to transfer into recipient cells inducing broad phenotypic changes [58]. In addition, exosomes are pleiotropic in nature and are capable of producing multiple signal transduction cascades. It is also evident that homeostasis relies heavily upon effective cell-to-cell communication (Fig. 1). Exosomes from distinctive stem cells and from non-stem cells at different developmental stages may carry unique, timely bio-information. Since communication depends on an efficient crosstalk between the exosome and the target cell, it is of import that exosomes provide paracrine communication via direct receptor stimulation of target cells and the horizontal transfer of genetic material, for cells to receive this bio-information.

Using exosomes for cancer therapy may provide many benefits. First, due to their small size and higher relative internal pressure (Laplace Law), they penetrate the cell membrane without difficulty to deliver their cargo. Second, exosomes can be engineered with a specific drug or molecule inserted, causing a desired effect on the target cell. Third, exosome bioavailability and function is generally maintained once injected into the body due to the protective effects of the phospholipid bilayer. A report in 2015, tested the properties of miR-134 derived from exosomes of patients with triple-negative breast cancer (TNBC) and detailed the therapeutic potential of miR-134 as a tumor suppressor [59]. It was confirmed that miR-134 reduced TNBC aggression and enhanced drug sensitivity of the cancer cells. MiR-134, in combination with a chemotherapy drug, cisplatin, increased cellular sensitivity to cisplatin-induced apoptosis. These data demonstrated that miR-134 may have therapeutic potential as an onco-suppressor when used in combination with chemotherapy drugs. Micro-RNAs (miRNAs) may provide a rapid method of regulating gene expression in cancer cells. These miRNAs are candidates for post-transcriptional regulation and induction of epigenetic changes in the recipient cells, and their effects may be further enhanced by photo-biomodulation, mitochondrial cofactors, oxygen and ketogenic diet [60].

In addition to the therapeutic potential of exosomes as anti-tumor bio-delivery systems, an emerging restorative property of these vesicles is the reprogramming of terminally differentiated cancer cells (Fig. 1). This approach refers to any intervention that transforms oncogenic cells back into benign cells, through the re-programming of their epigenetic profile [10, 61]. In recent years exosome-mediated re-programming has been explored as a promising platform with the potential to revert cancer cells to normal cellular function. Competing technologies include transcription factor-mediated and small molecule-mediated reprogramming, but while effective, both have clinical disadvantages in delivery method inefficiency and safety concerns [62–64]. Conversely, exosomes have the capacity to inhibit cellular proliferation of cancer cells by altering epigenetic marks associated with oncogenesis and have been demonstrated to inhibit cancer progression with targeted specificity and minimal toxicity to healthy cells [65, 66]. Exosomes contain a number of cell differentiation factors which are critical for regulating cell fate. For example, exosomes derived from mesenchymal stromal cells (MSCs) possess differentiation factors, such as stem cell differentiation stage factors (SCDSF), that have been demonstrated to inhibit tumor growth when delivered to patients with intermediate-advanced hepatocellular carcinoma (HCC) [67]. The authors noted that the treatment was easy to apply and resulted in negligible side-effects for patients, making it a promising therapeutic avenue to pursue given that conventional cancer therapies have often severe side-effects.

### Exosome derivatives and their potential as cancer therapies

#### Embryonic stem cell factors and cancer

Embryonic stem cells (ESC) are pluripotent cells that can be differentiated into many cell types, when provided with the appropriate signaling molecules. This feature, together with the unlimited capacity for proliferation of these immortal cells makes them an interesting therapeutic tool in regenerative medicine. In addition, to their differentiation and proliferation potential, evidence suggests that ESCs may poses the ability to directly inhibit the development of cancer during the embryonic stage due to stage-specific factors present in embryonic cells [68]. Experimental attempts to induce malignant transformation in early-stage animal embryos has been fruitless, while post-organogenesis, the fetus becomes vulnerable to carcinogens [68]. Furthermore, when tumor cells are implanted into embryos prior to the period of organogenesis, they become differentiated. However, when these tumor cells are implanted into adult animals, they promote tumor development [69]. These data indicate that there may be specific factors present in the embryonic microenvironment that are critical in determining oncogenesis.

Further evidence of this came in 2019 when researchers observed that factors from Zebrafish embryos, during specific developmental phases, can inhibit breast cancer cells. The group found that these factors were able to retard cell proliferation, induce apoptosis, and prevent invasion and migration of cancer cells [70]. These apoptosis-inducing effects and the ability to inhibit cell proliferation have also been reported when using Zebrafish embryonic cells to antagonize colon cancer cells [71]. A number of factors have been identified from Zebrafish embryos following the initiation of stem cell differentiation [69]. These proteins include multiple isoforms of the yolk protein, vitellogenin, and heat shock proteins (e.g. HSP8 and HSP70), which play important roles in the immune response and regulation of mitochondrial metabolism. These proteins are also involved in many pathways, such as cell-cycle regulation, protein trafficking, chaperoning, protein synthesis and protein degradation. These proteins act on the transcriptional activation of p53 [72] and post-translational modifications of retinoblastoma (pRb), which are able to reprogram, or redirect cancer cells toward an apoptotic fate [73]. In addition, it was observed during a further study that exosomes from umbilical cord derived MSCs inhibited growth and induced apoptosis of glioblastoma cells, in vitro [74, 75]. It can be deduced that embryonic stem cells possess the necessary signals to induce therapeutic differentiation in cancer cells. The embryonic environment has the intrinsic ability to epigenetically program or reprogram cellular states during development or malignancy. It is now becoming increasingly clear that cancer cells seem to retain a level of plasticity to respond to embryonic differentiation signals that may induce growth arrest and loss of the malignant phenotype. This growth arrest is associated with the upregulation of cell cycle inhibitors and key signaling pathways involved in cell proliferation. Moreover, MSC exosomes have been shown to express functional respiratory complexes, which may promote aerobic ATP synthesis restoration in cancer cells [75].

### Mesenchymal stromal cell-derived exosomes and cancer

Mesenchymal stromal cells (MSCs) are found in multiple tissues and preside over tissue homeostasis and differentiation. Research has shown that they can also help in tumor suppression due to the specific exosomes they secrete. Exosomes derived from MSCs contain multiple cargoes and proteins that may control different metabolic pathways in malignant cells. In 2012, researchers evaluated whether exosomes from MSCs from adult human bone marrow could inhibit in vivo and in vitro growth of multiple tumors. The study revealed that exosomes secreted from MSCs of human bone marrow inhibited cell-cycle progression and induced apoptosis in various types of cancer cells including hepatoma, ovarian tumor and Kaposi’s sarcoma [66].

#### MSC-derived exosomes from umbilical cord

MSCs are found within, and can be isolated from, multiple tissues of the body, such as adipose tissue, bone marrow, umbilical cord and amniotic fluid. These cells have the capacity to differentiate into various cellular lineages (in vitro), have immunomodulatory capabilities and demonstrate antimicrobial properties [61]. MSC’s have the capacity for self-renewal while maintaining multipotency, thereby retaining a long-term stem-like phonotype, in vitro [61].

Human umbilical cord stem cells (hUCSCs) are unique cells derived from Wharton’s jelly, which possess interesting advantages for regenerative and anti-cancer therapy [61]. The hUCSCs derived from Wharton’s jelly, compared to other MSCs, possess a unique transcriptome that demonstrates expression of pro-apoptotic and anti-cancer genes including POUF1, NANOG, SOX2 and LIN28, and the IL12A cytokine associated with inducing apoptosis [76]. In 2009, a study suggested that intravenous administration of naïve hUCSCs for three weeks significantly attenuated growth of human breast carcinoma in a xenotransplant rat model [77], while another study observed that exosomes from hUCSCs inhibited growth and induced apoptosis of glioblastoma cells, in vitro [78]. The authors of these studies concluded that, when co-cultured with cancer cells, hUCSCs, produced factors that attenuated cancer cell growth. This conclusion was supported by a 2012 study that evaluated the role of human Wharton’s jelly stem cell (hWJSC) extracts in three different cancer cell types [78]. In these experiments all cancer cell lines exhibited cell shrinkage and blebbing. Taken together, these findings may open a door to possible new therapeutic strategies for cancer.

#### Amniotic fluid-derived extracellular vesicles and cancer

A subclass of EVs can be obtained from amniotic fluid which, with the appropriate method of isolation, can provide an even greater yield compared to other sources. As we discussed previously there exist several methods for the isolation, purification and characterization of EV products. A recent publication by Dr. Ian White, describes an amniotic fluid EV derivative referred to as "cytosomes". These cytosomes are harvested from full-term, healthy amniotic fluid collected by AATB-accredited, Food and Drug Administration (FDA) approved cord blood banks under consent [11]. Critically, Dr. White and his team have perfected methods to isolate, purify and store these cytosomes for use in research and clinical trials. Others, including Antounians et. al, have reported the importance of the method of isolation in performance of amniotic fluid-derived EVs [79]. They investigated several isolation techniques and reported variations in protein content, purity and expression of EV markers, as well as EV-mediated cell survival [79]. Overall, amniotic fluid-derived EVs, if isolated appropriately possess anti-apoptotic, pro-angiogenic, and immune-modulatory properties, which can be influential in the treatment of certain cancers.

## Conclusions

Exosomes are nanoparticles critical for intercellular communication. Due to their ability to pass bio-information between cells, they can perform multiple functions in the body. Cancer treatments are in need of improvement in broad areas including effectiveness and specificity. Recent research has demonstrated that exosomes can be a very sharp tool in the anti-cancer toolbox. They carry multiple cargoes (proteins, miRNA mRNA, lipid and complex sugars), with effects that result in a multitude of specific actions within cells. Exosomes contain immunogenic molecules that are required to stimulate T-cells in order to induce apoptosis in cancer cells. Exosomes can also be loaded with potent factors, from different populations of regenerative cells, that can result in differentiation or apoptosis of cancer cells. The communicative ability of exosomes makes them a chief bio-molecule primed as a natural therapeutic agent, since they can cause beneficial phenotypic modifications within all cell populations. In addition to their therapeutic potential as an anti-cancer treatment, exosomes also have the ability to modulate the immune function and thereby have the capacity to be used as a treatment to alleviate pain and other chronic illnesses. Currently, there is a high incidence of opioid addiction within the US as a result of its overly prescribed use as a pain reliever. Growing data supports the idea that exosomal therapy can be used as an alternative treatment for inflammation and pain, resulting in a decrease in prescribed opioids and potentially lowering the incidence of opioid addiction.

The therapeutic ability of exosomes to fight cancer is very promising. Due to exosomes being relatively easy to use, specific and non-toxic, they have a future as a targeted drug delivery nanoplatform. Further studies are needed to explore the successful modification of exosomes as engineered, customizable therapeutic systems. Moreover, the cellular source of exosomes to be used to engineer these biological drug delivery systems requires further exploration. A better understanding of the functional differences and biological characteristics of exosomes based on cellular origin is critical for their clinical application.

Cancer cells seem not to be irretrievably locked in the malignant state, in the presence of embryonic differentiation control systems, they could be returned to normal. This reprogramming of the cancer cell epigenome may be accomplished by modifying DNA methylation and histone acetylation of the silenced tumor suppressor genes, which would then be delivered to the target cells by exosomes. However, more research is needed to fully understand the exosome derivative to select for specific effectiveness, which depends on the type of treatment required. We need to better understand the physiological effects of different exosome populations during the various developmental stages and of different tissues delineations. Further research is warranted to learn more about the composition of these nanovesicles in order to expose their fullest potential as a cancer treatment option.

## Data Availability

Not applicable.
